# Cardiovascular Outcomes in Advanced Maternal Age Delivering Women. Clinical Review and Medico-Legal Issues

**DOI:** 10.3390/medicina55100658

**Published:** 2019-09-29

**Authors:** Daniele De Viti, Antonio Malvasi, Francesco Busardò, Renata Beck, Simona Zaami, Enrico Marinelli

**Affiliations:** 1 Department of Cardiology, Santa Maria Hospital, GVM Care & Research, 70124 Bari, Italy; 2 Department of Obstetrics and Gynecology, Santa Maria Hospital, GVM Care & Research, 70124 Bari, Italy; 3 Section of Legal Medicine, Università Politecnica delle Marche, 60120 Ancona, Italy; 4 Department of Anesthesia and Analgesia, Santa Maria Hospital, GVM Care & Research, 70124 Bari, Italy; 5 Unit of Forensic Toxicology (UoFT), Department of Anatomical, Histological, Forensic and Orthopedic Sciences, Sapienza University, 00161 Rome, Italy

**Keywords:** pregnancy, advanced maternal age, cardiovascular outcomes, peripartum cardiomyopathies (PPCM), hypertension, diabetes, arrhythmias

## Abstract

*Background and objecives*: Adverse cardiovascular outcomes during pregnancy have increased over the past few decades, with increased numbers of women delivering later in their reproductive life. Other factors include higher rates of female obesity, diabetes, hypertension, cardiovascular diseases and assisted reproductive technology, which has extended fertility. Those at risk require extensive prenatal maternal screening, constant pregnancy supervising, monitoring during labor, delivery and puerperium and careful anesthetic evaluation during delivery. *Materials and Methods*: The present review reports the relevant information available on cardiovascular outcomes in advanced maternal age delivering women and related medico-legal issues. The search was performed on Pubmed, Cochrane, Semantic Scholar, Medline and Embase databases, accessed by Ovid, including among others the terms “cardiomyopathy”, “ischaemic heart disease”, “arrhythmias”, “hypertension”, “peripartum period”, “diabetes”, “advanced maternal age” “anesthesia”, “maternal morbidity and mortality” and “litigation”. *Results*: To the extent that underestimating risk factors for peripartum cardiomyopathy (PPCM) can adversely impact maternal and fetal outcomes, the legal implications of misdiagnosis or mismanagement can result in high compensatory damages. Substantial indemnity payments drive up costs of insurance coverage. *Conclusions*: Multidisciplinary approaches are necessary from obstetricians, cardiologists, anesthesiologists and perinatologists for pregnancy monitoring and delivery outcomes.

## 1. Introduction

Advanced maternal age (AMA) is defined as childbearing in a woman over 35 years of age or greater at the estimated date of delivery [1,2,3]. Motherhood at or beyond the edge of reproductive age is a new aspect of what clinicians previously referred to as pregnancy in the “older gravida” [4]. Recently, as a consequence of the combined effect of social changes and medical progress, the prevalence of AMA has increased, especially in high-income countries, and some women are even delaying childbirth until their forties [5,6,7].

International literature demonstrated that some cardiovascular risk factors such as hypertensive disorders (including chronic hypertension, gestational hypertension, preeclampsia and eclampsia) or glycometabolic disorders and the relative cardiovascular consequences such as peripartum cardiomyopathy (PPCM), ischaemic cardiac events and arrhythmias up to sudden cardiac death may occur more frequently in older mothers as a result of previously developed diseases (Table 1) [8,9,10,11,12].

In addition, it has been also suggested that women with very advanced maternal age (vAMA), defined as 45 or older, are even at higher risk of adverse pregnancy outcomes than women with AMA [13]. In this concern, although fertility declines with age, assisted reproductive technology has given this group of women an increased opportunity to become pregnant [11,12,13].

The present review reports internationally available relevant information on cardiovascular outcomes in AMA delivering women, as well as the related adverse legal developments occurring in related negative maternal and fetal outcomes.

## 2. Materials and Methods

A literature search was performed on the multidisciplinary research databases Pubmed, Cochrane, Semantic Scholar, Medline and Embase, accessed by Ovid, to identify all the relevant articles up to April 2019. The search included the terms “cardiomyopathy”, “ischaemic heart disease”, “arrhythmias”, “hypertension”, “peripartum period”, “diabetes”, “advanced maternal age”, “preeclampsia”, “eclampsia”, “anesthesia”, “maternal morbidity and mortality” and “litigation”. The search was limited to articles in English and to studies performed on humans. Two independent reviewers screened titles, abstracts, and full article studies to identify relevant studies. Prospective and retrospective studies including long-term follow-up reported outcomes (≥1 year) that included cardiovascular outcomes and all-cause mortality were selected. Case reports, case series and sample size ≤10 were excluded. Whenever available, the following information was extracted from each study: demographic characteristics such as age, ethnicity, comorbidities, the duration of follow-up and the reported mortality. The primary outcomes of interest were cardiovascular events including hypertension, preeclampsia, new onset of cardiomyopathy, arrhythmias together with diabetes mellitus and all-cause mortality. Mother’s age at delivery was the independent variable of interest.

With respect to medico-legal issues related to misdiagnosis of PPCM, court cases in which peripartum cardiomyopathy had occurred but did not directly bring about adverse consequences were disregarded. Legal databases were delved into, and five legal decisions were selected on the basis of their relevance in terms of presenting peripartum cardiomyopathy as the determining factor as to the patient’s adverse clinical outcome. It was then narrowed down to those cases in which the final decision as well as the scope of compensatory damages awarded were divulged.

## 3. Results

One hundred and fifty six articles were initially found, but after excluding duplicates, one hundred and twenty six studies were considered and reviewed. Unfortunately, ninety five studies were excluded because no classification based on maternal age was reported. Finally, eleven articles including quantitative data of prevalence of cardiovascular outcomes relative to the AMA vs <35 years old pregnancy population were included in this review [10,14,15,16,17,18,19,20,21,22,23]. Other studies in which quantitative data were not available to support our arguments were only considered to support quantitative data in the discussion section.

Table 2 and Table 3 summarize the demographic characteristics and cardiovascular outcomes together with the risk factor incidence of severe maternal morbidity due to cardiovascular disease in both <35 years and ≥35 years of age (AMA) delivering mothers in the considered studies. Unfortunately, in the reported studies, the statistical evaluation of any significant difference in outcomes between <35 and >35 years delivering women was not provided. Therefore, only the prevalence of the clinical characteristics under consideration could be listed. However, the trend in the different prevalence of cardiovascular complications of the two groups can be observed.

There was an elevated incidence of hypertension and glycometabolic disorders in the group of AMA with respect to that of the <35 years old group. The prevalence of preeclampsia ranged from 0.9% to 25.8% in the group of AMA and from 0.7% to 20.8% in the group of <35 years of age. Gestational diabetes prevalence ranged from 2.5% to 14.8% of AMA women and from 1.5% to 5.7% in the <35 years old group.

The incidence of PPCM in the group of AMA ranged from 0.008% to 0.4%, whereas in the group of <35 years, this ranged from 0.005% to 0.2% [19,20,21,22,23]. The prevalence of arrhythmias ranged from 0.005% to 0.19% in the AMA group vs a range from 0.005% to 0.05% in that of <35 years [19,21]. A diagnosis of acute myocardial infarction was present at the discharge in 0.004–0.049% of the AMA group and in 0.004–0.016% of the <35 years group [19,20]. The prevalence of aortic dissection was two times greater in the group of AMA [19]. There were two cases of sudden cardiac death in the AMA group due to ventricular fibrillation in the contest of cardiogenic shock, and asystole occurred one day after an elective cesarean section [22].

Claims involving the misdiagnosis of PPCM are reported in judicial databases Lexis, Justia and Leagle, as illustrated in Table 3 and Table 4. Furthermore, it is noteworthy that in cases of peripartum cardiomyopathy-related complications, misdiagnosis and failure to take appropriate steps in a timely fashion were both observed as major causes of litigation and extremely substantial compensatory damages were awarded to aggrieved patients.

## 4. Discussion

Pregnancy at advanced maternal age (over 35 years) has increased in many high income countries over the past several decades [24,25,26], with reported recent rates of, e.g., 9.1% in the United States (US) [27] and 28.1% in Japan [28].

As a consequence of that, it has been highlighted that AMA delivering women present a higher risk of developing an adverse cardiovascular outcome as compared to their younger counterparts [29].

In relation to this concern, the most frequent cardiovascular outcome during pregnancy is represented by hypertension disorders, which are strongly related to the advanced age of the pregnant woman. Indeed, increasing age is associated also with glucose intolerance due to a reduction in insulin sensitivity and abnormal lipid profile with increased levels of triglycerides and cholesterol [5].

Nationwide data from the USA have shown that the risk of pre-eclampsia does not appear to be affected by age before 35 years, but increases thereafter by 30% per year [30], and the risk for late-onset pre-eclampsia increases by 4% for every year over the age of 32 years [31].

In agreement with that, a large population-based study in the Canadian region of Ontario found that mothers at age >43 years had a higher risk of developing an adverse event cardiovascular composite outcome consisting of preeclampsia and gestational diabetes [14].

More recently (2013–2014), in Texas, USA, a higher frequency (*p* < 0.05) of gestational diabetes, pregestational diabetes, chronic hypertension and pregnancy related hypertensive disorders was noted in the very advanced maternal age (vAMA) at childbirth (>45 years) as compared to the youngest maternal age group [32].

Similarly, in Japan, a cross sectional study showed that pregnant women aged 45 years and older had a 1.5–2 fold greater risk of experiencing maternal morbidity compared to younger women (aged 30–34) Specifially speaking of preeclampsia, the risk of this outcome, including severe preeclampsia, was also especially increased among delivering women of 45 years and over [33]. Furthermore, the effect of maternal age on preeclampsia and severe preeclampsia were greater among multiparous women than in primiparous women, in contrast to what was previously reported in literature [34]. As both primiparity and advanced age are considered strong risk factors for preeclampsia, primiparous women of advanced age may be more likely to receive medications as compared to multiparous women [35]. The above reported Japanese study suggests that such practice should be considered for not only primiparous women, but also for multiparous women of advanced age [15].

Similar results were also presented in a study based in the UK in 2013, in which it was established that significant contributions from maternal age between 35 and 39.9 years, maternal age of 40 years or more, weight, chronic hypertension, Type 1 diabetes, previous preeclampsia and family history of preeclampsia were provided in the prediction of preeclampsia and gestational diabetes [10].

Analogous results were exhibited in the Australian Registry of births: women of very advanced maternal age (vAMA) aged 45 years or older were characterized by an increased incidence of pregnancy complications such as preeclampsia, gestational diabetes, antepartum hemorrhage and caesarean section [18]. A trend of favorable outcomes, even at extremely advanced maternal age (50–65 years), was evident for healthy women who had been screened to exclude pre-existing disease.

In addition to diabetes and preeclampsia, as a result of the greater prevalence of hypertension and glicometabolic disorders related to advanced maternal age, the consequent development of cardiovascular adverse events such as PPCM, ischaemic events or arrythmias related to them has also increased. Indeed, two retrospective studies, carried out in the USA, the first between 1991 and 2003 and the second between 2006 and 2015, both observed severe maternal morbidity because of cardiac disease during delivery women aged of ≥45 years [36,37].

Specifically, from 2004 to 2011, in the USA the incidence of PPCM was highest among women aged 40 to 54 years (36.7 (95% CI 35.4 to 37.9) per 10,000 live births), with approximately 20% such patients also at risk of moderate to severe deterioration of left ventricular function, persisting after delivery in 20% to 50% of patients [38,39,40,41,42,43]. In addition, in a Netherlands prospective population based cohort study in the period 2004–2006, increasing prevalence of advanced maternal age and pre-existing hypertension constituted strong risk factors for the development of PPCM during pregnancy [19]. These results underline that AMA is a strong risk factor for the development of PPCM and this category of pregnant women has to be carefully evaluated and followed during pregnancy. 

Similarly, a large USA study during the period 2000–2012 found that women 41 to 50 years of age had overall greater frequency of any arrhythmia and greater increase in any arrhythmia over time (199 per 100,000 and 162% increase) compared with women 18 to 30 years of age (55 per 100,000 and 58% increase) [21]. Supraventricular tachycardia was the most frequent sustained arrhythmia, and any type of arrhythmias were more frequent in black women than in the white women. Arrhythmias in pregnancy were associated with elevated odds ratio for mortality and maternal or fetal complications. Furthermore, in this analysis the authors report an increase in the frequency of arrhythmias by 58% during the period 2000–2012. A contributor to the increased frequency of arrhytmias could be the increase in maternal age [21]. The incidence of arrhythmias, especially atrial arrhytmias, normally increases with age and the presence of arrhythmias during pregnancy makes management of the patient extremely difficult because of restrictions in the use of drugs due to their potential fetal toxicity. 

With respect to myocardial infarction, in the USA within the period 2000–2002, among the pregnancy-related discharges, there were 859 cases of acute myocardial infarction [44]. Of these, 626 cases (73%) occurred during pregnancy, and 233 (27%) occurred postpartum, requiring readmission to the hospital. The mean age was 33 years for those with acute myocardial infarction and 27 years for those without it. The odds of acute myocardial infarction were 30-fold higher for women aged 40 years and older than for women <20 years of age. This shows that AMA is correlated with a high risk of acute myocardial infarction and, furthermore, that pregnant women in advanced age should be followed even after pregnancy.

Comparably, a large retrospective cohort in Canada showed a significantly increased myocardial infarction rate from 0.00 to 0.02 per 1000 deliveries between the triennium ending in 1993 and in 2000, giving a relative risk (RR) of 3.70 (95% CI 1.21–11.35) [45].

As a consequence of increased cardiovascular complications in advanced maternal age pregnancy, the related mortality due to cardiovascular events in these pregnant women has also risen concurrently. Although the overall risk of dying from pregnancy complications is generally low, some women are at a higher risk than others. The percentage of pregnancy-related deaths in the USA during 2011–2014 caused by cardiovascular diseases was 15.2% of cases with cardiomyopathy in 10.3% cases [46,47,48,49,50,51]. Proportionate mortality by cause of death did not change considerably at the USA population level in 2011–2013, even compared with data before the 2006–2010 period. The contribution of traditional causes of pregnancy-related mortality (hemorrhage, hypertensive disorders of pregnancy, thromboembolism) continued to decline, whereas that of cardiovascular and other medical conditions increased. For both 2006–2010 and 2011–2013 periods, cardiovascular conditions, including cardiomyopathy, were responsible for approximately 26% all pregnancy-related deaths [20]. Correspondingly, in a registry of pregnancy-related cardiovascular deaths in California, USA, among 2,741,220 women who gave birth, 864 died while pregnant or within 1 year of pregnancy; 257 deaths were deemed pregnancy related, and of these, 64 (25%) were attributed to cardiovascular disease [52], whereas a review of pregnancy-related maternal mortality in Wisconsin (2006‒2010) confirmed that age was a possible indicator of the risk of maternal mortality, with the youngest (<20 years) and oldest (>35 years) mothers exhibiting a higher PPCM than women between 20 and 34 years of age [23]. 

Data regarding maternal mortality by cardiovascular outcomes in countries of the world other than the USA are in general agreement with those of previous studies [34,35,53,54,55,56,57]. Specifically concerning Europe, there was no change in the overall maternal death rate in the UK between 2010–12 and 2013–15, being 8.76 per 100,000. There has been a significant 23% decrease in indirect maternal mortality since 2010–12 (95% CI 1–40%), primarily due to a decrease in influenza deaths and deaths from indirect causes of maternal sepsis. Cardiac disease remains the leading cause of indirect maternal death during or up to six weeks after the end of pregnancy with a rate of 2.34 per 100,000 maternities (data from 1997–1999 reported 1.6 per 100,000 maternities). The rates of maternal mortality varied by age, and the rate of maternal mortality was higher among older women (72% of women who died during or up to one year after pregnancy were >35years and 34% had BMI >30) [58,59]_._ Similarly, in the Netherlands for the period 2004–2006, the incidence of severe maternal morbidity due to cardiovascular disease was 2.3 per 10,000 deliveries. The maternal mortality rate from cardiovascular disease was 3.0 per 100,000 deliveries. The case fatality rate in women with severe maternal morbidity due to cardiovascular disease was 13% with the case fatality rate highest in aortic dissection (83%). Pre-existing acquired or congenital heart disease was identified in 34% of women. Thirty-one percent of women were of advanced maternal age (>35 years of age) and 5 percent above 40 years of age. Finally, the increase in maternal deaths from cardiovascular disease in the Netherlands is similar to findings in the UK [19]. These suggestive data regarding mortality during pregnancy should lead us to a careful clinical evaluation of pregnant women of advanced maternal age, or of those presenting other well-known risk factors such as preeclampsia, African descent or multiple pregnancy [60].

The awareness of the risk of cardiovascular outcomes in delivering AMA women has been reflected in the guidelines on the management of women with cardiac disease [61,62,63]. These documents all agree that in AMA women preconception counselling during pregnancy is essential and should be based on past history, underlying condition, and on assessment of the current physical state. Addressing cardiovascular disease in pregnancy requires early identification of disease, ideally before pregnancy, and continuous, risk-appropriate specialist care and follow-up throughout the pregnancy, on one hand to ensure the health of the mother and the unborn child and on the other to avoid legal issues related to eventual negative outcomes.

There are some important medico-legal implications related to early diagnosis and treatment of cardiovascular complications during pregnancy. First, it should be considered that the onset of cardiomyopathy can easily be masked and missed. Pregnant women most commonly can present dyspnea, dizziness, atypical chest pain, cough, neck vein distension, fatigue, and peripheral edema [64]. PPCM is a diagnosis of exclusion; its definitive diagnosis depends on echocardiographic identification, although in PPCM the electrocardiogram may be normal. Given that the disease is rare and the symptoms can be masked and missed, echocardiography is essential for diagnosis [65]. Hence, the prognosis is the best when PPCM is diagnosed and early treated [66]. No randomized clinical trials have been done to evaluate advanced age pregnant women with elevated cardiovascular risk. The management of this new population of women needs an accurate risk stratification with a thorough cardiovascular history and examination, 12 lead ECG and transthoracic echocardiogram. Echocardiographic evaluation should be performed during the last month of pregnancy and in the first five months postpartum in all patient with at least two risk factors including advanced maternal age (>35 years), history of hypertensive disorders or metabolic disorders, obesity (BMI >30), especially if associated with polycystic ovarian syndrome and with history of fertility problems, and in multiple pregnancies due to previous infertility treatment. Clinicians should be prepared for the challenges and potential cardiovascular complications related to patients who are epidemiologically different than those seen in the past. In this context, various guidelines provide disparate recommendations regarding starting antihypertensive therapy [67].

A final consideration for these kind of pregnant women concerns delivery management since these AMA women have to be considered risk patients and have to be treated by a multidisciplinary medical team. A cesarean section is recommended to be induced in general anesthesia with continuous haemodynamics semi invasive monitoring of blood pressure, heart rate, stroke volume, cardiac output or cardiac index and systemic vascular resistance, avoiding drugs that produce myocardial depression, maintaining normovolaemia and preventing increased afterload (Vigileo, NICaS, ICG) [67,68,69,70]. If the neuraxial technique of anesthesia is practiced, low drug doses and combined spinal-epidural anesthesia have to be used [71]. Actually, neuraxial anesthesia may induce hypotension due to marked peripheral vasodilatation and reduced venous return only partially compensated by a rise in the cardiac index (CI) [72]. During spinal anesthesia, blood pressure is inversely related to the dose of local anesthetics, which can thus play a key role in hemodynamic effects [73,74]. Therefore, the dose of local anesthetics has been widely studied to find out the right matching between blood pressure reduction and perioperative analgesia quality [75,76,77]. For instance, it was recently suggested to use a combined spinal and epidural (CSE) technique when the dose of levobupivacaine is < effective dose 95% (ED95), to perform a prompt rescue in case of insufficient anesthesia [78]. The CSE technique involves both spinal and epidural drug administration for pain relief combining a rapid and reliable onset of profound analgesia, resulting from spinal injection and using, in cases of necessity, further analgesia, through the epidural catheter [79]. Maternal cardiac function monitoring, which more directly reflects uteroplacental perfusion changes, would be crucial in the hemodynamic management of parturients undergoing a cesarean section to evaluate placental perfusion [80]. Even if a cesarean section is a matter of minutes in experienced hands, maternal hemodynamic instability may occur, especially in preeclamptic women; actually, on one side regional anesthesia may induce hypotension due to marked peripheral vasodilatation and reduced venous return only partially compensated by CI [72]. Indeed, the reported incidence of hypotension after neuraxial block varies between 7.4% and 74.1% [72]. Traditionally, three methods have been used for the prevention and management of spinal anesthesia-induced hypotension: fluid therapy, vasopressors and local anesthetic dosage [81]. Large volume preloading has been widely used as a strategy to prevent hypotension, [82] but this strategy cannot be used in patients with cardiac pathology. There is widespread variation in the choice of administration of vasopressors in obstetric anesthesia, with ephedrine and phenylephrine acknowledged by the National Institute for Health and Care Excellence to be equally efficacious to counteract hypotension in healthy parturients, although phenylephrine is claimed to be more preferable because of improved fetal acid-base status [83]. Furthermore, maternal hemodynamic changes during spinal anesthesia for cesarean section are traditionally evaluated by heart rate and non-invasive blood pressure, but recent studies addressed the importance of cardiac output monitoring in the assessment of maternal hemodynamic stability [84,85,86].

Finally, modern neuraxial analgesia favors initiation and maintenance of analgesia with low-dose local anesthesia and opioid solutions to minimize risks of local anesthetic systemic toxicity (unintentional intravascular injection) or high- or total-spinal anesthesia (unintentional intrathecal injection) [87]. These low-dose strategies also minimize hemodynamic effects and placental drug transfer [88]. Dilute local anesthetics reduce the risk of severe hypotension. Combining local anesthetic with lipid soluble opioid allows for profound visceral and somatic analgesia. The synergy between opioid and local anesthetic medications allows dose-reduction of both drugs, minimizing side-effects [89].

## 5. Conclusion

Pregnancy at advanced maternal age has become more common over the last decades. There is an increasing prevalence of women who delay childbirth for various reasons worldwide. Women at advanced maternal age have increased risk of adverse pregnancy cardiovascular outcomes. Cardiovascular disease is a rare cause of severe maternal morbidity with a high case fatality rate, especially in the case of aortic dissection. Most cardiac disorders in pregnancy, delivery or puerperium develop in women without pre-existing cardiac disease. The data presented in this review should increase awareness of the occurrence of severe maternal morbidity from cardiovascular disease and will lead to adequate risk assessment and timely referral of women at risk. However, some studies concluded that there is no definitive medical reason for excluding very AMA women from attempting pregnancy on the basis of age alone, since there is a trend of favorable outcomes, even at extremely advanced maternal age (50–65 years), for healthy women who have been screened to exclude pre-existing disease [12]. Addressing cardiovascular disease in pregnancy requires early identification of disease, ideally before pregnancy, and continuous, risk-appropriate specialist care and follow-up throughout the pregnancy. Women with pre-existing cardiac disease should be referred for preconceptional advice, optimise preconceptional health and stay within scope throughout pregnancy in a specialized centre.

It is necessary to raise awareness that underestimating PPCM may give rise to majorly catastrophic maternal and fetal outcomes when misdiagnosis or mismanagement of such conditions occur, as well as adverse legal outcomes resulting in extremely high compensatory damages. PPCM may lead to maternal death, permanent, severe disability and, last but not least, expose doctors and health care facilities to substantial indemnity payments, which may in turn make it nearly impossible to gain insurance coverage.

## Figures and Tables

**Table 1:** Risk factors for adverse cardiovascular outcomes during pregnancy.

hypertensive disorders	chronic hypertension gestational hypertension previous pre-eclampsia family history of pre-eclampsia
glycometabolic disorders:	pre-existing diabetes mellitus gestational diabetes mellitus
advanced maternal age	age ≥ 35 years
very advanced maternal age ≥ 45	age ≥ 45 years
Body Mass Index (BMI) abnormalities	Underweight Overweight Obesity
cigarettes smoke	

**Table 2:** Demographic and cardiovascular aspects and incidence of severe maternal morbidity due to cardiovascular disease in advanced maternal age (AMA) pregnancy women.

Author Year of the Study Country	Khalil et al. 2013 UK [10]	Wu et al. 2013 UK [14]	Ogawa et al. 2005–2011 Japan [15]	Tseng et al. 2010–2014 Taiwan [16]	Kahveci et al. 2011–2015 Turkey [17]	Carolan et al. 2005–2006 Australia [18]
No. patients	20,386	87,179	16.1236	190	486	217
Mean age (±SD), years		37.5 ± 4.0			37.5 ± 3.9	>45 *
BMI(kg/m^2^) mean ± SD		25.9 ± 6.2	21.5 ± 3.2		30.1 ± 4.1	
Underweight		2698 (3.1%)				
Normal weight		36,597 (42%)				
Overweight		19,925 (23.1%)				
Obese		14,667 (14.9%)				
Gestational HT	481 (2.3%)		6608 (4.1%)		37 (7.6%)	
Pre-existing HT		1486 (1.7%)	2153 (1.3%)			6 (2.8%)
Preeclampsia	507 (2.5%)	751 (0.9%)	7239 (4.5%)	49 (25.8%)	39 (8.0%)	10 (4.6%)
Gestational DM	516 (2.5%)	7849 (9%)		26 (13.7%)	72 (14.8%)	21 (9.7%)

* Mean age was not reported; BMI, body mass index (Kg/m^2^); HT, hypertension; DM, diabetes mellitus; SD, standard deviation.

**Table 3:** Demographic and cardiovascular aspects and incidence of severe maternal morbidity due to cardiovascular disease in <35 years old pregnant women.

Author Year of the StudyCountry	Khalil et al. 2013 UK [10]	Wu et al. 2012–2015 Canada [14]	Ogawa et al. 2005–2011 Japan [15]	Tseng et al. 2010–2014 Taiwan [16]	Kahveci et al. 2011–2015 Turkey [17]	Carolan et al. 2005–2006 Australia [18]
No of patients	55,772	298,844	204,181	293	471	48,909
Mean age (±SD), years		28.7 ± 3.7			27.6 ± 4.2	30–34 *
BMI (kg/m^2^) (mean ± SD)		25.3 ± 6.2	21.1 ± 2.8		28.7 ± 4.1	
Underweight		15,876 (5.3%)				
Normal weight		134,814 (45.1%)				
Overweight		62.058 (20.8%)				
Obese		46.129 (15.4%)				
Gestational HT	1326 (2.4%)				20 (4.2%)	
Pre-existing HT		2025 (0.7%)	1241 (0.6%)			558 (1.1%)
Preeclampsia	1191 (2.2%)	2215 (0.7%)	7136 (3.5%)	61 (20.8%)	22 (4.6%)	1237 (2.5%)
Gestational DM	839 (1.5%)	13,618 (4.5%)	5184 (2.5%)	16 (5.5%)	27 (5.7%)	2425 (5%)

* Mean age was not reported; BMI, body mass index (Kg/m^2^); HT, hypertension; DM, diabetes mellitus.

**Table 4:** Peripartum cardiomyopathy-related malpractice lawsuits.

Drawn from Judicial Databases (Lexis, Justia, Leagle)		
Patient, Age Date of Delivery	Clinical Developments	Damage Suffered and Litigation Outcome
L.P. 33-year-old patient. August 2000, MN, USA.	L.P. was admitted to ER on August 28th 2000, hypertensive and tachycardic and later admitted to labor and delivery. According to experts, the doctors repeatedly failed to investigate the patient’s tachycardia, hypertension, tachypnea and shortness of breath. Plus, despite abnormal vital signs, the nurses failed to adequately assess maternal status to appropriately respond to adverse changes in maternal and fetal signs, and to initiate appropriate safeguards. After delivery, L.P. went into a cardiac arrest, experiencing seizure-like activity. Her chest X-ray showed diffuse bilateral airspace disease most likely due to edema. Further evaluation, including echocardiogram findings, was consistent with peripartum cardiomyopathy.	L.P. is permanently disabled from the hypoxic ischemic (damage to cells in the brain and spinal cord from inadequate oxygen) brain injury she suffered. She lives in a nursing home, dependent on care givers for all basic activities, including eating, toileting, dressing and mobility. In the fall of 2005, the patient (through M.P., her husband and guardian) received $2.618.000 as a medical malpractice settlement ^(*)^.
L.T. 25-year-old patient. February 2005, Newport News, VA, USA.	The patient gave birth to her first child on February 27th. A few months later, she went to ER with chest pains and an X-ray revealed an enlarged heart. On four different occasions the patient was misdiagnosed with viral illnesses and sent home despite swelling of feet and ankles, chest pain, fatigue and enlarged heart, revealed by X-rays. Five days later, after her fourth trip to ER, she was finally diagnosed with post-partum cardiomyopathy and congestive heart failure.	Efforts to treat the patient failed. She received a heart transplant and will likely need a second heart transplant later on. She will have to take anti-rejection drugs to prevent her immune system from attacking her new organ for the rest of her life. After just three hours of deliberation, a jury returned with a verdict of $4 million ^(**)^.
G.C. 41-year-old patient. December 2007, Colquitt County, GA, USA	G.C. had high blood pressure during her pregnancy. Twice that month, defendant Dr. A. hospitalized her for observation and to bring down her blood pressure. She delivered her baby by c-section on December 17th. On December 19th she went home. Her blood pressure was 130/90. Two days later, she wasn’t well, experiencing extreme swelling. Her feet were extremely swollen. A nurse took her blood pressure twice and got readings of 170/88 and 168/90, yet the blood pressure readings were not recorded on her chart, and the doctor did not examine her.	The patient went into a coma after being released from the hospital with high blood pressure two days after giving birth by caesarian section. The woman emerged from the 45-day coma with permanent brain damage and can no longer walk without assistance, is legally blind, and her hands are “cupped” so that she cannot feed herself. It was later found she was suffering from peripartum cardiomyopathy, presenting telltale signs: blood pressure going up, difficulty breathing, edema or swelling. Colquitt County Superior Court jury awarded the patient $5 million ^(***)^.
S.E. May 2009, DeKalb County, GA, USA.	S.E.’s blood pressure problems had initially been treated, but in the hours before her death, her condition became more precarious with low oxygen levels and blood-gas levels joining her complaints that she was short of breath. Despite this, medical records showed staff did not take S.E.’s vital signs for three hours before she went into fatal cardiac arrest. S.E. died of heart failure due to cardiomyopathy three days after being admitted to the hospital for preeclampsia and ultimately giving birth to her daughter.	The verdict capped a nearly three-week trial in which it was claimed S.E. died due to the failure of her healthcare team to detect or treat her deteriorating condition. Jurors awarded more than $3 million to the patient’s family, splitting fault between two nurses who treated her, but cleared her doctor of negligence ^(****)^.
E.R. 36-year-old patient. December 2011, Chicago, IL, USA.	E.R.’s blood pressure began to rise after delivering twins at Ingalls Memorial Hospital, according to the family’s attorney. Nurses alerted E.R.’s doctor when her blood pressure rose to dangerous levels two days later, but they failed to administer the drugs he prescribed. After visiting her baby, E.R.’s heart rate rose and her oxygen saturation dangerously plummeted. Against the hospital’s policy, nurses again failed to give her the prescribed drugs or alert a rapid response team.	The patient went into cardiac arrest causing severe brain damage. Two months later on February 6th 2012, she passed away of heart failure from postpartum cardiomyopathy. The suit, which was settled in Cook County Circuit Court, awarded E.R.’s family $4 million ^(*****)^.

* Robis & Kaplan LLP. K.P. v. Hospital/Docs. M.P., individually, and as guardian for his wife L.P., and their son, Date of Disposition: Fall, 2005. ** Schwaner Injury Law. Virginia Case Illustrates Seriousness of Condition. May 2015. *** Tucker K.H. Meeks law. Daily Report October 19th, 2012. **** Crisco A. Courtroom View Network. Feb 2nd, 2017. ***** NBC Chicago. Family of Woman Who Died After Giving Birth at Suburban Hospital Awarded $4M. Published May 9, 2018.
